# Critical Gaps in Medical Research Reporting by Online News Media

**DOI:** 10.7759/cureus.57457

**Published:** 2024-04-02

**Authors:** Thomas F Heston

**Affiliations:** 1 Medical Education and Clinical Sciences, Washington State University, Spokane, USA; 2 Family Medicine, University of Washington, Spokane, USA

**Keywords:** medical news reporting, conflicts of interest, research integrity, transparency, online news media

## Abstract

Background: The integrity of medical research reporting in online news publications is crucial for informed healthcare decisions and public health discourse. However, omissions, lack of transparency, and the rapid spread of misinformation on digital and social media platforms can lead to an incomplete or inaccurate understanding of research findings. This study aims to analyze the fidelity of online news in reporting medical research findings, focusing on conflicts of interest, study limitations, statistical data, and research conclusions.

Methods: Fifty randomized controlled trials published in major medical journals and their corresponding news reports were evaluated for the inclusion of conflicts of interest, study limitations, and inferential statistics in the news reports. The alignment of conclusions was evaluated. A binomial test with a Bonferroni correction was used to assess the inclusion rate of these variables against a 90% threshold.

Results: Conflicts of interest were reported in 10 (20%) of news reports, study limitations in 14 (28%), and inferential statistics in 19 (38%). These rates were significantly lower than the 90% threshold (p<0.001). Research conclusions aligned in 43 (86%) cases, which was not significantly different from 90% (p=0.230). Misaligned conclusions resulted from overstating claims.

Conclusion: Significant gaps exist in the reporting of critical contextual information in medical news articles. Adopting a structured reporting format could enhance the quality and transparency of medical research communication. Collaboration among journalists, news organizations, and medical researchers is crucial for establishing and promoting best practices, fostering informed public discourse, and better health outcomes.

## Introduction

The dissemination of medical research findings through online news publications plays a crucial role in shaping healthcare decisions and informing public health discourse. However, even in legitimate news sources, the reporting of medical research is susceptible to omissions and a lack of transparency, which can lead to an incomplete understanding of the research findings [1]. The rise of digital and social media platforms has amplified this challenge, as these platforms can rapidly disseminate false claims without stringent verification processes, significantly influencing public health perceptions and behaviors. For example, health misinformation on social media has been linked to reduced compliance with public health guidelines, such as vaccine uptake [2,3], and to the amplification of an infodemic, a mixture of misinformation and true information about the origins and alternative cures of disease [4].

While considerable research has examined the impact of misinformation and fake news on social media, a focused investigation into the reporting practices of legitimate online medical news sources remains underexplored. The rapid evolution of digital media platforms has significantly altered the landscape of information dissemination, necessitating a closer look at the transparency and completeness of these online news outlets. 

This study evaluated online news reports compared to peer-reviewed medical research. Key inclusion criteria for readers to reasonably and accurately evaluate the medical news article were assessed.

## Materials and methods

The evaluation focused on four critical aspects of reporting: the mention of conflicts of interest (COIs) by study authors, the acknowledgment of study limitations, the inclusion of inferential statistical data (either p-values or confidence intervals with effect sizes), and the alignment of news report conclusions with those of the original research articles. These four variables were all binomial, categorized as yes (included in the news article) or no (not included in the news article). An inclusion rate of 90% for these four factors was set for sample size determination. This high but not perfect inclusion rate was meant to account for unintentional errors while aiming for an achievable high reporting integrity and transparency standard. A 90% target inclusion rate was considered reasonable given that since 2017, PubMed has included COIs below the abstract when supplied by the publisher [5]. The null hypothesis was that each variable would be present in at least 90% of the news reports, and the alternative hypothesis was that each variable would be present in less than 90% of the news reports.

Medical articles for evaluation were obtained from the PubMed database using the following strategy: ((((("The New England Journal of Medicine"[Journal]) OR ("JAMA"[Journal])) OR ("BMJ (Clinical Research ed.)"[Journal])) OR ("Annals of Internal Medicine"[Journal])) OR ("Lancet (London, England)"[Journal])) OR ("Nature medicine"[Journal]) AND ffrft[Filter] AND randomizedcontrolledtrial[Filter]

Journals were restricted to the *New England Journal of Medicine*, *JAMA*, *BMJ*, the *Annals of Internal Medicine*, *Lancet*, or *Nature Medicine*. These were chosen due to their high profile and thus increased likelihood of being covered by the news media.

Results were filtered to only include articles with free full-text availability. Also the results were filtered to only include randomized controlled trials.

The sample size was determined using several methods: (a) a power analysis was conducted using a one-sample test of proportions. For a 99% level of confidence and an expected proportion of 90% with a margin of error of 15%, a sample size of 27 would be required [6]; (b) to detect an effect size of 15% or greater from the null hypothesis of 90% using a two-sided test, an alpha of 0.05, a power of 80%, a sample size of 41 would be required [7,8]; and (c) finally, a common rule of thumb for sample size determination is to have 5 to 10 samples per variable [9]. Given these results, it was determined that a sample size of 50 would be reasonable and appropriate. 

Statistical analyses using IBM SPSS Statistics for Windows, Version 29 (Released 2023; IBM Corp., Armonk, New York) employed a binomial test of the null hypothesis. Given that an inclusion rate of over 90% would support the null hypothesis, a one-tailed p-value was utilized to determine if the inclusion rate for each variable was under 90%. The Bonferroni correction, which adjusts the significance level for multiple comparisons to reduce the likelihood of Type I errors, was used to determine the significance of p-values [10]. The SAMPL guidelines for health research reporting were followed [11].

Recognizing the potential sensitivity of the data and the importance of unbiased analysis, we anonymized the identities of the research articles and the news organizations. This approach was fundamental not only to prevent any undue focus on specific entities but also to maintain an objective stance that fosters a constructive dialogue about improving medical research reporting without targeting any specific organization. By anonymizing the data, we aim to highlight systemic trends and opportunities for enhancement rather than attributing shortcomings to individual publishers. This ethical consideration underscores our commitment to a respectful and responsible examination of the media landscape, contributing to a broader understanding and trust in medical science communication.

The dataset supporting our findings is accessible on the Zenodo repository, which offers transparency and enables further research in this area [12].

## Results

A total of 50 clinical research trials and their corresponding news reports were analyzed. The 50 news reports came from a total of 34 unique news organizations. The clinical research trials were all from 2023-2024.

Conflicts of interest (COIs) were mentioned in 10 articles (20% ± 5.8%, p < 0.001), indicating a significant underreporting compared to the 90% threshold. Study limitations were acknowledged in 14 articles (28% ± 6.5%, p < 0.001), also significantly lower than the 90% threshold. Inferential statistics, such as p-values or confidence intervals with effect sizes, were included in 19 articles (38% ± 7.0%, p < 0.001), again falling short of the 90% threshold. The news report agreed with the medical trial’s findings in 43 out of 50 cases (86% ± 5.0%), which is not significantly different from the null hypothesis of 90% (p = 0.240). However, if a higher standard of 95% agreement were set, then this rate of 86% would be determined to be significantly lower (p = 0.009) (Table 1).

**Table 1:** The inclusion rate of reporting criteria in news reports

Reporting Criteria	Number of Reports (%)	95% Confidence Interval	p-Value
COIs	10 (20%)	14.2 - 25.8%	< 0.001
Study limitations	14 (28%)	21.5 - 34.5%	< 0.001
Inferential statistics	19 (38%)	31.0 - 45.0%	< 0.001
Aligned conclusions	43 (86%)	81.0 - 91.0%	0.230

In all seven cases where the conclusions did not match, the reason was overhyping results by making unqualified claims not found in the research trial. The specific reasons for a mismatch of conclusions were as follows. (1) One news report only mentioned part of the conclusion indicating positive results and did not mention that the results were negative in the other arm of the trial. (2) Another news report expanded the finding of decreased resource utilization to include that patients received improved care when this was not proven and not one of the study’s outcomes. (3) A third mismatch occurred when the medical article showed findings restricted to an age group, but the news report indicated it was applicable to all age groups. (4) A fourth news report agreed with the article’s conclusion that a supplement “might” reduce cardiovascular events. Still, the report's title stated unequivocally that the supplement reduced cardiovascular events. (5) One news report stated that the new intervention completely eliminated the need for maintenance medical therapy. In contrast, the research determined that the intervention reduced but did not eliminate the need for ongoing maintenance medical therapy. (6) One medical research article found only that recurrence-free survival was improved with the new therapy; the news report stated that not only was recurrence-free survival improved, but there was also a reduction in deaths. (7) The final discordant study was due to the news report stating that the increased number of deaths in the intervention arm was unlikely due to the intervention. The research article, however, was much more cautious, stating that ongoing research into this finding was necessary.

Only four news reports included all four reporting criteria, and three did not include any of the reporting criteria analyzed.

## Discussion

This study reveals significant gaps in reporting critical contextual information in online medical news articles, such as COIs, study limitations, and inferential statistics. While research conclusions were generally conveyed accurately, underreporting these key elements raises concerns about the transparency and credibility of medical research communication. When news reports disagreed with the medical research, it was invariably due to overstating research findings by the news reports.

These findings have important societal implications. The low rates of reporting COIs, study limitations, and inferential statistics decrease readers' ability to properly understand and evaluate medical research. Without access to this contextual information, readers may overestimate the significance or applicability of research findings, leading to potential misinterpretations and the spread of misinformation [13]. Incomplete reporting of medical research can also have serious ramifications for public health outcomes. Omitting relevant information, such as COIs, hinders readers' ability to gauge a study's possible weaknesses, given that COIs have been shown to affect study results [14]. Additionally, the lack of inclusion of statistical analyses and a discussion of study limitations can prevent readers from fully evaluating the significance and generalizability of the research findings.

This study reinforces findings from previous research. One study reporting on 500 health news stories from major US news organizations found that most of these stories failed to adequately address costs, harms, benefits, the quality of the evidence, and alternative options [15]. Another study found that "spin" in press releases and news reports was related to the presence of "spin" in the abstract of peer-reviewed articles of randomized controlled trials (RCTs). This "spin" led to an overestimation of the benefit of the experimental treatment in 27% of reports based on the press release and 24% of reports based on the news item, compared to the full-text peer-reviewed article [16]. Similarly, another study evaluated 1889 health news stories and found that most stories failed to adequately address costs, harms, benefits, evidence quality, and alternative options when covering healthcare products and procedures [13]. Another study looking at pharmaceutical research found significant shortcomings, with 53% of news reports failing to mention potential harms and 70% not mentioning costs. This study also found that the COIs of experts quoted in news reports were disclosed only 39% of the time [17]. 

These findings underscore the need for more comprehensive and balanced reporting of medical research by the news media, as inadequate coverage can lead to misinformed healthcare consumers and decision-makers. However, efforts to combat misinformation through individualized solutions, such as labeling fake news sources and fact-checking, have significant limitations. They can exacerbate the problem of misinformation by failing to address the underlying systemic issues in journalism, tech platform dominance, and governmental policies [18].

To address these challenges, one potential solution would be the adoption of structured reporting formats for medical news articles, similar to those utilized in a standard clinical trial abstract and manuscript. Such structured reporting has been shown to improve the communication of medical information. For example, a study of 330 radiology reports found that referring physicians were more satisfied with structured reports and that the structured reports had greater clarity [19]. While not a uniform requirement, many imaging societies recommend structured reporting of scan results, including the American Society of Nuclear Cardiology [20] and the American College of Radiology [21]. Similarly, a structured format for medical research articles is recommended. For example, the International Committee of Medical Journal Editors recommends structured abstracts and a standardized overall structure for medical research articles [22]. 

One recommendation for news reporting is to follow standardized inclusion criteria when reporting on medical research. While news reports may utilize a narrative style, the contents would still follow a standardized flow. Specifically, the establishment of best practices in medical news reporting might call for the routine inclusion of background information, study methods, main results with statistical analyses, a discussion of implications and limitations, a conclusion that accurately reflects the research findings, and finally, a disclosure of COIs. 

While our study provides valuable insights, it is essential to acknowledge its limitations. Our focus on major medical journals and English-language publications may not fully represent the global landscape of medical news reporting. This study only looked at randomized clinical trials published in major medical journals in 2023 and 2024. Future research should expand the scope of analysis to include a broader array of sources and languages, and a wider variety of research types, providing a more comprehensive understanding of the challenges and opportunities for improving medical research communication worldwide. Despite these limitations, this study makes significant contributions to evaluating online misinformation. By focusing on clear and quantifiable components, medical news reporting was objectively assessed, and key areas for improvement were identified. Furthermore, the findings provide a compelling case for adopting standardized reporting to improve online communication of medical research. These findings also lay the groundwork for future research and interventions to promote accurate, transparent, and trustworthy communication of scientific knowledge to the public.

## Conclusions

This study reveals significant gaps in the reporting of medical research by online news organizations, particularly regarding transparency about COIs, discussion of study limitations, and the inclusion of relevant statistical data. Despite the encouraging agreement on research conclusions, there is a pressing need for improved reporting standards. Implementing structured reporting formats, similar to those used in clinical trial abstracts and manuscripts could enhance the quality and transparency of medical research communication. Collaboration among journalists, news organizations, and medical researchers is crucial to achieve this. By working together to establish and promote best practices, these stakeholders can foster a more informed public discourse on health and science topics, ultimately contributing to better health outcomes and decision-making.
